# Could dementia be detected from UK primary care patients’ records by simple automated methods earlier than by the treating physician? A retrospective case-control study

**DOI:** 10.12688/wellcomeopenres.15903.1

**Published:** 2020-06-08

**Authors:** Elizabeth Ford, Johannes Starlinger, Philip Rooney, Seb Oliver, Sube Banerjee, Harm van Marwijk, Jackie Cassell

**Affiliations:** 1 Department of Primary Care and Public Health, Brighton and Sussex Medical School, Watson Building, Village Way, Falmer, Brighton, BN1 9PH, UK; 2 Department of Computer Science, Humboldt University of Berlin, Rudower Chaussee 25, Berlin, 12489, Germany; 3 Department of Physics and Astronomy, University of Sussex, Brighton, BN1 9RQ, UK; 4 Faculty of Health, University of Plymouth, Drake Circus, Plymouth, Devon, PL4 8AA, UK

**Keywords:** Dementia, diagnosis, primary care, electronic patient records, early detection, machine learning.

## Abstract

**Background:** Timely diagnosis of dementia is a policy priority in the United Kingdom (UK). Primary care physicians receive incentives to diagnose dementia; however, 33% of patients are still not receiving a diagnosis. We explored automating early detection of dementia using data from patients’ electronic health records (EHRs). We investigated: a) how early a machine-learning model could accurately identify dementia before the physician; b) if models could be tuned for dementia subtype; and c) what the best clinical features were for achieving detection.

**Methods:** Using EHRs from Clinical Practice Research Datalink in a case-control design, we selected patients aged >65y with a diagnosis of dementia recorded 2000-2012 (cases) and matched them 1:1 to controls; we also identified subsets of Alzheimer’s and vascular dementia patients. Using 77 coded concepts recorded in the 5 years before diagnosis, we trained random forest classifiers, and evaluated models using Area Under the Receiver Operating Characteristic Curve (AUC). We examined models by year prior to diagnosis, subtype, and the most important features contributing to classification.

**Results:** 95,202 patients (median age 83y; 64.8% female) were included (50% dementia cases). Classification of dementia cases and controls was poor 2-5 years prior to physician-recorded diagnosis (AUC range 0.55-0.65) but good in the year before (AUC: 0.84). Features indicating increasing cognitive and physical frailty dominated models 2-5 years before diagnosis; in the final year, initiation of the dementia diagnostic pathway (symptoms, screening and referral) explained the sudden increase in accuracy. No substantial differences were seen between all-cause dementia and subtypes.

**Conclusions:** Automated detection of dementia earlier than the treating physician may be problematic, if using only primary care data. Future work should investigate more complex modelling, benefits of linking multiple sources of healthcare data and monitoring devices, or contextualising the algorithm to those cases that the GP would need to investigate.

## Introduction

Dementia is a global public health challenge and is one of the most common and serious disorders in the elderly population. The condition is characterized by progressive but often slow decline in memory, reasoning, communication and the ability to carry out daily activities. These symptoms can be caused by a number of illnesses that lead to structural and chemical changes in the brain, and death of brain tissue ^1^. Currently there are no treatments which are curative for dementia; however, a timely diagnosis of dementia can still be valuable particularly for younger patients and their families, as they can be offered a range of supportive or therapeutic interventions, inform themselves about the condition, and plan for their future financial and care needs, which may help them maximize their quality of life ^2^. For example, there is weak evidence that an early diagnosis followed by treatment with cholinesterase inhibitors may delay admission to institutionalised care ^3^, although current available treatments do not impact long-term prognosis.

The United Kingdom (UK) Government published a National Dementia Strategy in 2009 in which earlier diagnosis was one of the key aims ^4^. This was followed by the Prime Minister’s Challenge on dementia ^5^ and the general practice focussed dementia identification scheme ^6^. These initiatives have brought the estimated rate of diagnosis up from 50% to around 67% ^7^. Still, at the current time, one third of patients with dementia will not receive a recorded diagnosis for their condition in the UK’s National Health Service (NHS) ^7^, and many patients still receive their diagnosis late in the disease course when many opportunities for improving quality of life may have passed.

In the UK NHS, 98% of the population is registered with general practitioner (GP) clinics, which provide the majority of community-based generalist healthcare ^8^. The early recognition of dementia, initiation of the diagnosis pathway, and follow-up medical care for people with dementia in the UK occurs in general practice, and GPs receive financial incentives for maintaining dementia registers and providing care.

All the information about a patient’s interactions with the general practice clinic, and with some other services within the NHS, is noted in an electronic record; each piece of clinical information entered also has a time stamp. This means that the patient’s record is a longitudinal account of their healthcare history. These longtitudinal healthcare accounts may differ systematically between patients who are developing dementia and those who are not, in the few years before dementia is identified. This has been found in studies which have manually examined patient notes. These studies found that cognitive symptoms, contact with social care professionals, unpredictable consulting patterns, increased attendance, levels of carer involvement, and changes to gait were higher in patients in the few years preceding the dementia diagnosis, compared to patients who were not developing dementia ^9, 10^.

In the UK, the government curates large datasets of NHS patient records. One such dataset of GP patient records is the Clinical Practice Research Datalink (CPRD), which GPs can opt to contribute to, and which holds data on over seven million current patients ^11, 12^. These databases form huge resources for health research, especially as the data contained in them goes beyond a list of clinical diagnoses, and includes social, administrative and lifestyle information, as well as tests performed and referrals made. With the time stamps in the data, the development of diseases, their determinants, and their outcomes can be tracked over time.

These records are used by medical researchers for studies on the distribution of disease in the population ^13– 15^, risk factors for disease ^16– 19^, and for examining drug safety in a community population ^20, 21^. Databases like CPRD could provide a valuable resource for understanding the onset and early presentation of disorders like dementia, and may provide a rich data source for creating early detection algorithms that can subsequently be developed into tools for automated decision or diagnostic support in primary care, which could help GPs to discuss, detect and label dementia in a more timely way, over and above current detection rates, if clinically indicated.

While recent reviews of dementia risk prediction models show a large body of research on methods to predict likelihood of dementia at a future point ^22, 23^, we found few studies that aimed to model automated methods to detect current, early dementia, especially in primary care or using only primary care data ^24, 25^. So far, there has been little investigation of how accurate an early detection model using only primary care data could be to pick up dementia, or how much earlier than current diagnosis by GPs a tool could discriminate well between patients who go on to be diagnosed and those who do not. The aim of this study was to explore different facets of the hypothesis that GP patient record data could be used to create an early detection tool for dementia. We aimed to ascertain whether patients with dementia could be discriminated from matched control patients, prior to recorded diagnosis, using only their recorded primary care data, and if so:

1) How early this discrimination could be achieved;
2) Whether better discrimination could be achieved for dementia subtypes (Alzheimer’s and vascular dementia) based on different presentations, risk factors or symptoms patterns;
3) What the most important features would be for achieving good discrimination at each of a number of time points prior to diagnosis and for each subtype.

## Methods

### Ethical statement

This study was approved by the Independent Scientific Advisory Committee at the Medicines and Healthcare Products Regulatory Authority, UK [15_111_R]. Pseudonymised data is collected by CPRD for future research purposes with an opt-in mechanism (consent) at the GP level, and an opt-out mechanism at the patient level; thus, patients can state their dissent for their data to be used for secondary purposes. Individual consent from patients is not required for this de-identified data re-use, the lawful basis for this data processing under the General Data Protection Regulation 2018 is legal obligation (direction from the Secretary of State for Health to extract data to carry out additional functions concerning: information functions, the information functions of any health or social care body and systems delivery functions) and a task in the public interest (management of health and social care systems).

### Data source

This study used data from the UK CPRD. CPRD was established in 1987, and now contains anonymized healthcare records from more than 19 million current and historic patients and represents 13% of the UK population at any one time ^12^. Patients are broadly representative of the UK general population in terms of age, sex and ethnicity. CPRD includes longitudinal observational data from GP electronic health record systems in primary care practices, including medical diagnoses (using Read codes), referrals to specialists and to secondary care, testing and interventional procedures conducted in primary care, lifestyle information (e.g. smoking, exercise) and drugs prescribed in primary care ^11^. Data are captured using a structured hierarchical vocabulary called Read codes; these were developed by a UK GP, Dr James Read, in the 1980s to facilitate standardized assessment and semantic disambiguation of patient conditions when using computers. They map to other nomenclatures such as International Classification of Diseases (ICD), SNOMED-CT, and International Classification of Primary Care (ICPC) codes. Each Read code represents a term or short phrase describing a health-related concept. There are over 200,000 different codes, which are sorted into categories (diagnoses, processes of care and medication) and subchapters ^26^. Each clinical entity is represented by an alphanumeric code and a Read term, which is the plain language description.

### Study population

We constructed a case-control dataset. For this project, CPRD extracted the full records of patients with dementia (cases), identified on the basis of a code list for dementia diagnostic codes (general dementia, vascular and Alzheimer’s dementia codes) developed using code lists from Russell *et al.* ^27^, and Rait *et al.* ^28^, and used in Ford *et al.* ^24^, (Appendix 1, see *Extended data* ^29^). Patients had to be 65 years or older, and had to have records available in CPRD for at least three years prior to the first diagnosis code for dementia, which was recorded between 2000 and 2012. All dementia patients meeting these criteria were extracted from the CPRD Gold database. Control patients were randomly sampled from patients who matched cases on year of birth, sex, and general practice, but had no dementia codes from the list anywhere in their record, and were also required to have at least three years of record prior to the matched “index date”. This resulted in not more than one match for each case; not all cases received a match. The entire available patient record was extracted for each patient. This resulted in 47,858 cases and 47,663 controls.

### Data management

The data were screened and processed using the following steps: firstly we removed cases who had no matched control (N = 195), then we removed the few controls who had a dementia code (N=7) and their matched case (N=7). Following slight adjustment of our dementia code list to eliminate a code indicating only delirium (not dementia), we then removed cases who had no diagnostic dementia code (N=55) and their matched control (N = 55). This resulted in 47,601 cases and 47,601 controls. Only data from within the period five years before the index date were used. All data more than 5 years before, or at any time after, the index date were discarded. This was to standardise the sample, as fewer patients had data in each year over 5 years.

### Sub-populations

For aim (2), tuning by dementia subtype, we specified three groups of patients:

1) All cause dementia; defined as all dementia cases, regardless of specificity of diagnosis (N = 47,601).

2) Alzheimer’s disease sub-group (N = 13,452); this was defined as cases with a code for Alzheimer’s disease; but no further codes for vascular dementia or any other specific dementia (e.g. Lewy Body), and no mixed Alzheimer’s and vascular. Cases were retained in the Alzheimer group if they had other unspecified codes for dementia e.g. “Senile Dementia Unspecified”. Each patient’s matched control was kept with them for analyses.

3) Vascular dementia sub-group (N = 10,870); this was defined as cases with a code for vascular dementia, but no evidence for Alzheimer’s disease, or mixed type. Cases were retained in the vascular group if they had other unspecified codes for dementia. Each patient’s matched control was kept with them for analyses.

There was no overlap between patients in the Alzheimer’s disease and Vascular dementia subgroups.

### Feature selection

Because of the volume of different Read codes, and the fact that there are usually many Read codes representing the same clinical concept, we defined a wide list of clinical concepts or features *a priori*. We used two methods for selecting likely features. These were: 1) a systematic review and meta-analysis of existing literature from primary care records research on dementia ^30^; and 2) a consultation of 21 GPs working as clinical tutors at the same institution as the authors, approached at two tutor meetings, with the following written question to provide clinical grounding: “ *Please could you list anything you can think of which may frequently be entered in the patient record up to 3 years before a dementia diagnosis (it does not have to be causal, just occur earlier in time than the diagnosis).”* Responses were then handwritten as open free text answers on a sheet of paper and answers from all respondents were collated into a spreadsheet. The most common responses were: fall (suggested by six GPs); depression/low mood (eight GPs); anxiety (four GPs); cerebrovascular accident/transient ischaemic attack (six GPs); high blood pressure (five GPs); forgetful (five GPs); problem with memory (seven GPs); did not attend code (six GPs) (for full data on responses see Appendix 3, *Extended data* ^29^). **

GP responses were mapped against clinical concepts found to be associated with dementia in the meta-analysis to create a final list of features for the model. Features were operationalised by lists of Read codes. The features ranged from known or long-term risk factors for dementia (such as diabetes, smoking, or history of stroke), to health events related to general decline in health, mobility or cognition (such as infections, falls, burns and wound dressings), to GP activity around the dementia diagnostic process (such as memory loss codes, cognitive screening tests, blood tests, and referrals). We discarded suggested features such as “repeat consultations on the same thing” which could not be operationalised using a code list. Read code lists were sought for these predictors from an online code list repository ^31^ and from emailing authors of reviewed studies. Where code lists for features were not readily available, new lists were drawn up using the CPRD medical dictionary application by author EF and checked by PR. This resulted in a total of 77 code lists. (Appendix 2, see *Extended data* ^29^). Code lists were matched to event-level patient data. The five-year run up period was then split into five year-long sections. Binary features were created for each one-year time period, indicating whether a patient had ≥1 instances of a code from each code list in that period. The creation of binary rather than count features is thought to reduce the effect of frequency of GP visits in the data ^25^.

### Analysis

Data were preprocessed and analysed using custom Perl scripts (Perl version 5.30) for data ingest, cleansing and reshaping, and Python scripts (Python version 3.7.4) employing the pandas, matplotlib and scikit-learn libraries for model training and evaluation, and plot generation (see *Code availability*) ^32^.

***Choice of algorithm.*** We chose a single method to run all analyses on particular subsets of the data by time, and by dementia type (we term these “models”), as our aims were not to assess the effectiveness of different machine learning approaches. Studies suggest random forest algorithms are at least as good as and possibly better than conventional methods such as logistic regression and Cox proportional hazards regression for complex clinical prediction models when binary variables are used ^24, 33, 34^. Our previous research suggested they performed similarly to neural networks and support vector machines on this data source ^24^. They have the additional advantage that the list of features that contribute to the final classification and their relative weights are readily available as a secondary output of the training procedure, giving an indication as to which elements of the patients’ EHRs contribute to the classification result most strongly. We used the Random Forest Classifier in scikit-learn, with the number of “trees” in the “forest” set at 100. The “feature-importances” attribute in scikit-learn produces a set of estimates of importance of each feature in the random forest model whose values are positive and in total, the estimates for all features sum to 1.0. A normalized estimate of the predictive power of each feature is calculated from the fraction of samples a feature contributes to (related to how high up each tree the feature appears), combined with the decrease in impurity from splitting the samples. The higher the value given to each feature, the more important it is to the prediction ^35^.

***Assessment of accuracy.*** Using a fixed random number seed to ensure the same split of patients for each model, the data were split at random into 67% for training and 33% for testing. Each model was assessed for its ability to classify dementia cases versus controls using the Area Under the Receiver Operating Characteristic Curve (AUC) where an AUC of 0.5 indicates performance at chance, and an AUC of 1.0 indicates perfect classification of cases and controls ^36, 37^. A value of 0.7–0.8 is considered acceptable, 0.8–0.9 is considered good or very good, and 0.9 and above is excellent ^36^.

***Fit of models to aims.*** To address aim (1), how early dementia can be detected by using data methods, the earliest point in time when detection of dementia could be achieved, models were run to examine discrimination in each of 5 years before diagnosis based on codes in that year and all previous years. In the Year -5 model, only data from 5 years before diagnosis was included. In the Year -4 model, all data available prior to the end of the 4 ^th^ year before diagnosis was included (year -5 and year -4). In the Year -3 model, data from years -3, -4 and -5 were included, for Year -2, data from years -2, -3, -4, and -5, and for Year -1, data from all years were included. The reasoning underpinning this approach was that we were looking to find the earliest time at which cases and controls could be well discriminated, using data which preceded that time period.

To address aim (2), to evaluate if models could be tuned to dementia subtypes, these same analysis models were run on the whole dataset (all cause dementia) and then on subsets of Alzheimer’s patients with their matched controls, and Vascular patients with their matched controls.

To address aim (3), to identify the most important features for achieving good discrimination at each of a number of time points, we generated lists of the features contributing most strongly to discrimination in each of these time periods and for each outcome (all cause dementia, Alzheimer’s and Vascular), and examined the estimates of importance generated by the software for each feature.

## Results

### Study population

95,202 patients were included in the final analyses, these comprised 33,502 men (35.2%) and 61,700 women (64.8%). Patients had a median age of 83 years at index date (range 65–110 years). All patients had at least three years’ worth of data, 90,351 patients (97.0%) had at least four years and 87,876 patients (94.4%) at least five years.

For the 13,452 Alzheimer cases, there were 4,409 men (32.8%) and 9,043 women (67.2%); with median age of 82 years, (range 65 -104), these retained their original matched controls with the same characteristics. For the 10,870 vascular dementia cases, there were 4,415 men (40.6%) and 6,455 women (59.4%); the median age was 83 years (range 65 – 103), and the equivalent in their matched controls.

### Aim 1: When is the earliest that we can discriminate between cases and controls?

**Figure 1.**
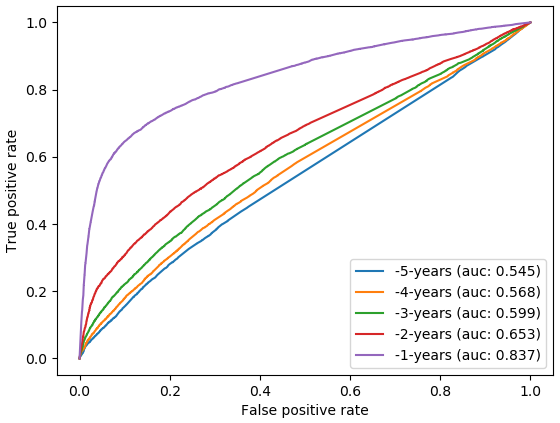
Receiver operating characteristic (ROC) curves for random forest classifiers -5 to -1 years before diagnosis for all-cause dementia. AUC, Area Under the ROC Curve.

Using the 5 years of analysis and all specified predictive features, we found that discrimination between cases and controls was poor (AUC range 0.55–0.65) in the 5 to 2 years prior to diagnosis, but showed slight but continuous improvement over this time. In the final year before diagnosis the model showed good discrimination (AUC: 0.84; Figure 1).

### Aim 2: Can the model be tuned to achieve better discrimination for subtypes of Alzheimer’s disease or Vascular dementia?

**Figure 2.**
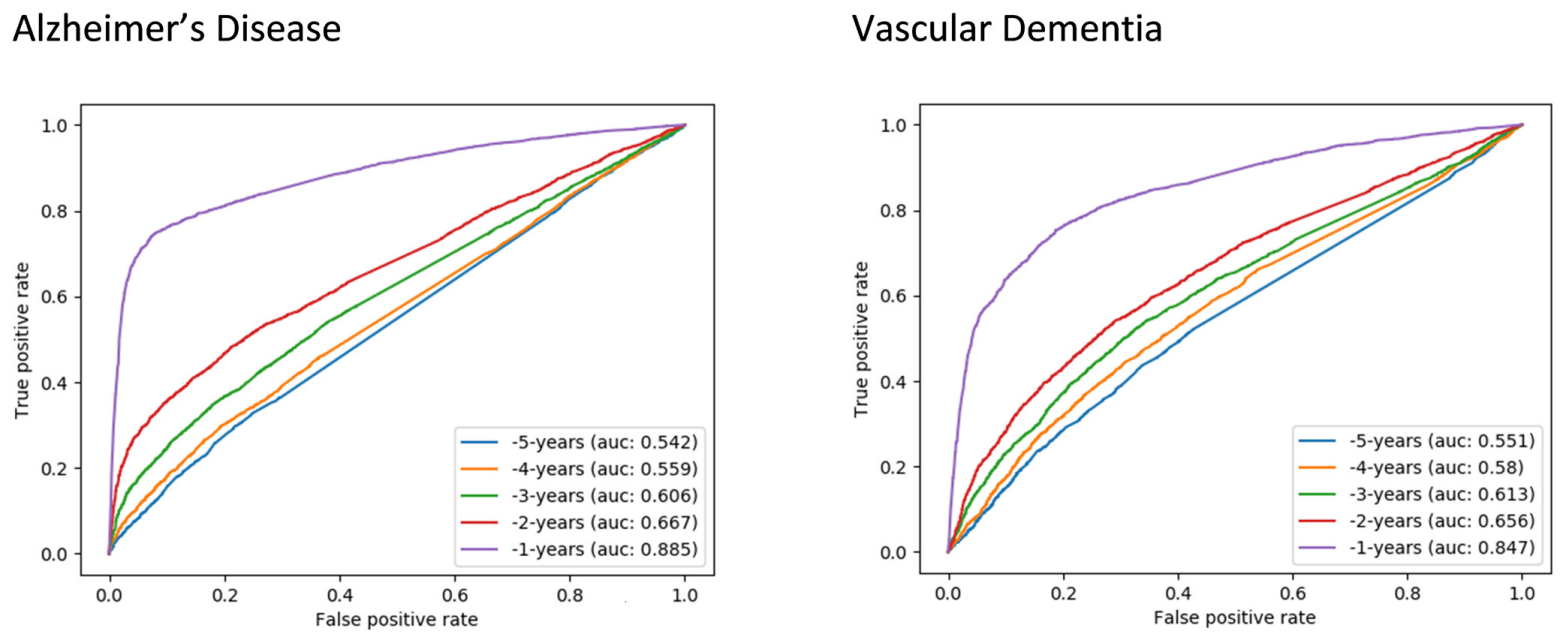
Receiver operating characteristic curves (ROC) for random forest classifiers for dementia subtypes: Alzheimer’s disease (left) and vascular dementia (right). AUC, Area Under the ROC Curve.

Using the same set of features, very similar findings as for Aim 1 were shown for the Alzheimer’s and Vascular subsets. In the final year before diagnosis the discrimination of the Alzheimer’s model was very good (AUC 0.89), and the Vascular model was also good (AUC 0.85). In years -2 to -5, the classifiers were poor to fair for both subsets (AUC 0.54–0.67) ( Figure 2).

### Aim 3: Examining the most important features for good discrimination

Inspecting the 15 most highly weighted features within each year block revealed that the increase in model performance over time was associated with a gradual shift in the types of codes recorded for patients over the time period examined ( Table 1). Five years prior to diagnosis long term risk factors and progressive indicators of frailty and ill health predominated. From 4 years before current diagnosis, memory loss codes contributed more and more to the model, and by 2 years before this feature dominated all the other features. Only in the last year preceding diagnosis did action towards diagnostic underpinning become apparent with cognitive screening tests and referrals for further assessment becoming the most predictive features. The features most highly weighted in the models for the subsets of Alzheimer’s and Vascular dementia patients showed largely similar results ( Table 2 and Table 3) although stroke and cardiovascular risk factors were more conspicuous among the most highly weighted predictors for Vascular dementia.

**Table 1.** Most highly weighted features over time (all cause dementia).

	Year -5	Year -4	Year -3	Year -2	Year -1
1	Infection (0.049)	Memory loss codes (0.052)	Memory loss codes (0.071)	Memory loss codes (0.104)	Memory loss codes (0.223)
2	Is a smoker (0.044)	Infections (0.046)	Is a smoker (0.041)	Is a smoker (0.037)	Referral to Psychiatrist Neurologist or Geriatrician (0.063)
3	Antidepressants (0.041)	Is a smoker (0.043)	Infections (0.039)	Dressing of Wound Burn or Ulcer (0.035)	Cognitive Screening (MMSE) (0.040)
4	Memory loss codes (0.038)	Visit to Emergency Dept (0.037)	Dressing of wound, burn or ulcer (0.038)	Hospital admission (0.035)	Antidepressants (0.025)
5	GP home visit (0.035)	Hospital admission (0.037)	Hospital admission (0.036)	Infection (0.034)	Did not attend code (0.023)
6	Dressing of wounds, burns or ulcer (0.035)	Dressing of wounds, burns or ulcer (0.035)	Urinary tract infection (0.034)	Visit to emergency dept (0.033)	Infections (0.023)
7	Hospital admission (0.035)	Urinary Tract Infection (0.034)	Antipsychotics (0.032)	Urinary tract infection (0.031)	Is a smoker (0.023)
8	Urinary Tract Infection (0.034)	Deafness (0.030)	Deafness (0.031)	Antipsychotics (0.030)	Hospital admission (0.022)
9	Visit to Emergency Dept (0.032)	GP Home visit (0.029)	Visit to Emergency Dept (0.030)	Deafness (0.030)	Visit to emergency dept (0.022)
10	Coronary Heart Disease (0.028)	Chronic kidney disease (0.028)	Chronic Kidney Disease (0.029)	Chronic Kidney disease (0.028)	GP Home visit (0.022)
11	Hypertension (0.028)	Antidepressants (0.028)	Hypertension (0.029)	GP Home Visit (0.026)	Urinary Tract infection (0.020)
12	Did not attend (0.026)	Antipsychotics (0.027)	GP Home visit (0.028)	Antidepressant (0.025)	Dressing of wound burn or ulcer (0.020)
13	Antipsychotics (0.026)	Hypertension (0.027)	Coronary Heart Disease (0.026)	Hypertension (0.024)	Referral to memory assessment service (0.020)
14	Z-drugs (for insomnia) (0.025)	Did not attend (0.027)	Did not attend (0.024)	Did not attend (0.022)	Constipation (0.020)
15	Deafness (0.024)	Coronary heart disease (0.026)	Constipation (0.022)	Coronary heart disease (0.021)	Had a fall (0.019)

*Key: Red – long standing condition or risk factor; Orange – could be associated with increasing frailty or prodromal dementia symptoms; Green – General Practitioner has detected dementia symptoms.* *Values for each predictor represent its relative contribution to the model compared to all other predictors (with all predictors together summing to 1.0, and a larger value being assigned to more important predictors; 77 predictors were included).*

**Table 2.** Most highly weighted features over time for Alzheimer’s disease.

	Year -5	Year -4	Year -3	Year -2	Year -1
1	GP Home Visit (0.053)	Memory loss codes (0.057)	Memory loss codes (0.080)	Memory loss codes (0.128)	Memory loss codes (0.309)
2	Is a smoker (0.049)	GP Home visit (0.050)	GP Home visit (0.044)	GP Home visit (0.041)	Referral to Psychiatrist Neurologist or Geriatrician (0.080)
3	Infections (0.044)	Infections (0.046)	Visit to Emergency Dept (0.042)	Visit to Emergency Dept (0.036)	Cognitive Screening (MMSE) (0.052)
4	Visit to Emergency Dept (0.043)	Visit to Emergency Dept (0.042)	Infections (0.038)	Dressing of wound, burns or ulcer (0.035)	Referral to memory assessment service (0.030)
5	Dressing of wounds, burns or ulcer (0.038)	Is a smoker (0.041)	Dressing of wound, burns or ulcer (0.036)	Is a smoker (0.034)	Antidepressants (0.021)
6	Antipsychotics (0.038)	Antipsychotics (0.038)	Hospital admission (0.036)	Infections (0.033)	Alzheimer-specific medication (0.021)
7	Memory Loss Codes (0.036)	Dressing of wounds, burns or ulcer (0.035)	Is a smoker (0.032)	Hospital admission (0.032)	Visit to Emergency Dept (0.019)
8	Hospital Admission (0.034)	Hospital admission (0.033)	Antipsychotics (0.035)	Antipsychotics (0.032)	Did not attend (0.019)
9	Antidepressants (0.033)	Deafness (0.030)	Urinary Tract Infection (0.033)	Deafness (0.029)	Infections (0.018)
10	Urinary tract infection (0.031)	Chronic kidney disease (0.030)	Deafness (0.030)	Urinary tract infection (0.028)	Hospital Admission (0.018)
11	Did not attend (0.031)	Did not attend (0.029)	Chronic Kidney Disease (0.027)	Did not attend (0.026)	GP home visit (0.017)
12	Z-drugs (for insomnia) (0.028)	Urinary Tract Infection (0.027)	Did not attend (0.027)	Chronic kidney disease (0.026)	Is a smoker (0.017)
13	Deafness (0.027)	Constipation (0.026)	Hypertension (0.025)	Constipation (0.024)	Cognitive Decline (0.017)
14	Chronic Kidney Disease (0.025)	Antidepressants (0.024)	Falls (0.023)	Hypertension (0.023)	Urinary Tract Infection (0.017)
15	Constipation (0.024)	Hypertension (0.023)	Constipation (0.022)	Antidepressants (0.022)	Dressing of wound, burn or ulcer (0.016)

*Key: Red – long standing condition or risk factor; Orange – could be associated with increasing frailty or prodromal dementia symptoms; Green – GP has detected dementia symptoms*. *Values for each predictor represent its relative contribution to the model compared to all other predictors (with all predictors together summing to 1.0, and a larger value being assigned to more important predictors; 77 predictors were included)*.

**Table 3.** Most highly weighted features over time for vascular dementia.

	Year -5	Year -4	Year -3	Year -2	Year -1
1	Infections (0.054)	Infections (0.049)	Infections (0.047)	Memory loss codes (0.069)	Memory loss codes (0.169)
2	Dressing of wound, burn or ulcer (0.041)	Hypertension (0.042)	Memory loss codes (0.045)	Infections (0.037)	Referral to Psychiatrist Neurologist or Geriatrician (0.070)
3	Antidepressants (0.039)	Dressing of wound, burn or ulcer (0.041)	Dressing of wound, burn or ulcer (0.037)	Dressing of wound, burn or ulcer (0.036)	Cognitive Screening (MMSE) (0.039)
4	GP Home visit (0.038)	Hospital admission (0.037)	Hypertension (0.037)	Stroke (0.035)	Stroke (0.037)
5	Did not attend (0.036)	GP home visit (0.035)	Hospital admission (0.036)	Hypertension (0.034)	GP Home visit (0.033)
6	Hospital admission (0.035)	Visit to emergency dept. (0.035)	Is a smoker (0.035)	GP home visit (0.033)	Antidepressants (0.029)
7	Visit to Emergency dept. (0.033)	Is a smoker (0.033)	Antipsychotics (0.033)	Hospital admission (0.033)	Did not attend (0.025)
8	Stroke (0.032)	Memory loss codes (0.032)	Visit to Emergency Dept. (0.033)	Did not attend (0.032)	Falls (0.025)
9	Urinary Tract Infection (0.031)	Antidepressants (0.032)	GP Home Visit (0.032)	Chronic Kidney disease (0.032)	Visit to emergency dept. (0.024)
10	Antipsychotics (0.031)	Did not attend (0.032)	Urinary Tract infection (0.032)	Visit to emergency dept (0.031)	Infections (0.024)
11	Deafness (0.030)	Urinary Tract Infection (0.031)	Stroke (0.032)	Is a smoker (0.031)	Is a smoker (0.023)
12	Is a smoker (0.030)	Antipsychotics (0.030)	Chronic kidney disease (0.031)	Urinary tract infection (0.029)	Dressing of wound burn or ulcer (0.022)
13	Hypertension (0.029)	Deafness (0.029)	Did not attend (0.030)	Deafness (0.029)	Hospital admission (0.022)
14	Coronary Heart Disease (0.027)	Stroke (0.029)	Deafness (0.030)	Antidepressants (0.029)	Hypertension (0.022)
15	Falls (0.026)	Chronic kidney disease (0.027)	Antidepressants (0.028)	Antipsychotics (0.028)	Third party consultation (0.021)

*Key: Red – long standing condition or risk factor; Orange – could be associated with increasing frailty or prodromal dementia symptoms; Green – General Practitioner has detected dementia symptoms.* *Values for each predictor represent its relative contribution to the model compared to all other predictors (with all predictors together summing to 1.0, and a larger value being assigned to more important predictors; 77 predictors were included).*

## Discussion

We found that models discriminating between dementia cases and controls in the five-year period before diagnosis, using only information from primary care EHRs, were most successful in the final year before diagnosis, producing very good discrimination. This aligns with clinical intuition. Models performed similarly in the sub-groups of Alzheimer’s disease and Vascular dementia patients. However, we noted that the type of predictors most highly weighted in the model changed over time prior to diagnosis. At 5 years before diagnosis, known clinical risk factors such as smoking and cardiovascular risk factors were predictive alongside indicators that the patient was experiencing increasing health events such as visiting the hospital. During years 4–2 before diagnosis, evidence of deterioration in general health, increase in frailty, and prodromal dementia symptoms were among the best predictors, which may be an indication of early symptoms of dementia becoming manifest, although memory loss symptoms were the top predictors in all these years. Finally, in the year before diagnosis, there was evidence of activity happening in primary care, which would lead the patient onto the pathway towards a dementia diagnosis, such as screening tests and referral. Good classification only became possible in this final year, when the patient was already being investigated for dementia symptoms. Life-time dementia risk factors, and other clinical events such as accidents or infections, were not sufficient to offer very good discrimination between potential dementia cases and controls on their own. We found no substantial differences between predictors for Alzheimer’s or Vascular sub populations compared to all cause dementia.

### Implications

The idea of using clinical decision support, populated only with routinely collected clinical data from primary care, with the aim of bringing forward dementia diagnosis to an earlier time point, seems challenging given these results. It would be interesting to discuss these findings further with local stakeholders to see whether this provides them with information that is helpful in clinical practice. Our findings suggest that while earlier risk factors, such as smoking or heart disease, may occur in higher rates in patients who go on to develop dementia, the differences in prevalence of these factors between individuals with dementia and those without may not be great enough, even when multiple conditions are combined, to discriminate between these populations. In addition, we found that our model only became accurate when evidence was accumulating in the record that the GP had themselves picked up on signs of dementia. This suggests that a simple binary classifier, using routinely collected clinical data from patient records, will struggle to outperform a clinician, given that these data are recorded by the clinician the algorithm is trying to support.

### Strengths and limitations

We used data from over 95,000 real life patients treated in the NHS, collected in real time. This seems an adequate sample size for our purposes, and was made up of all patients available in the CPRD database who met our criteria, thus selection effects should be low. The external validity to the real world of our data is also high. However, because of the fact that these were ‘real-life’ data, we know there may be misclassifications in the training data due to the fact that the data were not specifically collected for research but for care purposes. Their internal validity (or consistency across coders) may be low. However, we did not “clean” false negative cases out of our control sample. This is because we would have needed to use many of the features we used in our predictive model to identify these false negatives, thus leading to a form of incorporation bias. A further limitation is heterogeneity of clinic visits and timings of patients within the sample; for example, for those patients with very little data, our algorithm was unlikely make a good prediction, and this would be the same for an algorithm running in the clinic setting. It is also possible that control patients had less data or fewer visits, which could lead to bias in the model. Clinical coding in primary care, especially with an open and unstructured system like the Read codes (compared to the ICPC, for instance), necessarily picks up a lot of randomness ^38^.

Given that age is the biggest known risk factor for dementia, and this was effectively eliminated from the model by the matched design, it is likely that future versions of the model would be improved by using a cohort design and incorporating age as a potential feature. Additionally, sex may be a useful predictor, given that rates of dementia are around twice as high in women as in men. It is possible that other predictors that we did not include, such as medication prescriptions, would also contribute to better discrimination. We were additionally restricted to information which is routinely gathered in primary care. Known risk factors such as APOE-4 genotype, low educational attainment, levels of physical activity and social isolation, and other potential cognitive or neuro-psychiatric assessments are not well-captured in these records. Future studies that link research cohort and primary care data may be able to incorporate such additional risk factors and therefore improve the accuracy of predictive models.

A further limitation with our approach is that we only used data from the five years prior to diagnosis code or matched date. This may have reduced the contribution of long-term conditions as features in the model, given that these may have been coded in patients’ records prior to the study period. Previous research using primary care records, and dementia risk prediction tools have found that chronic conditions such as diabetes, cardiovascular disease and depression are all likely increase future risk of dementia ^23, 30^. Of note in our study, smoking was an important feature throughout all models, and stroke and hypertension were among the top predictors for vascular dementia, which would be expected; however, the more transient or newly experienced indicators of developing frailty dominated the models. Additionally, the consideration of the differences between vascular and Alzheimer’s dementia is limited by their commonly occurring together as a mixed dementia.

### Future research

While our model showed that there is some traction for this method perhaps in the year before diagnosis, it did not indicate a clear signal in primary care data that would advance the detection of dementia in the GP clinic to a point earlier in time; there are further avenues to explore. A recent policy report on dementia risk prediction models called for stratification of prediction models by different dementia subtypes, and also for modelling of change of behaviour or other features over time ^22^. Given that dementia is characterised by progressive onset, a potential future avenue for automated decision support may be to identify patterns of behaviour or increase in morbidity or frailty over time. This approach would be strengthened by the linkage of multiple sources of healthcare data, for example from both primary and secondary care, as well as from wearable or monitoring devices, and by exploring more complex machine learning methods, perhaps with several methods running in series or in parallel. While a model based on signs and symptoms may not tell a clinician anything they do not already know, a model based on picking out characteristic patterns that develop over time, identified from several data sources, such as changes in gait or frailty ^10^, may inform a clinician of an incipient problem that has been overlooked in a busy clinic.

## Conclusions

We aimed to ascertain if cases of dementia could be discriminated from controls prior to diagnosis, to examine how early this discrimination could be achieved, and to uncover the best features for achieving good discrimination. We found predictive features evolved over time and the best discrimination was clearly achieved in the year before diagnosis when the features suggested that GPs were already cognisant of symptoms of the condition. If automated early detection were to be developed from routine clinical data, and for it to have clinical utility, we suggest modelling characteristic patterns of dementia onset over time, possibly from multiple sources of linked data. Such patterns could be missed by clinicians. Such risk modelling work should be embedded in a culture of stakeholder engagement, where clinicians and patients are consulted about their preferences for such an approach and can guide its implementation.

## Data availability

### Underlying data

The patient data that support the findings of this study are available from Clinical Practice Research Datalink (CPRD; www.cprd.com) but restrictions apply to the availability of these data, which were used under license for the current study, and so are not publicly available. For re-using these data, an application must be made directly to CPRD. Instructions for how to submit an application and the conditions under which access will be granted are explained at https://www.cprd.com/research-applications.

### Extended data

Zenodo: Could dementia be detected from UK primary care patients' records by simple automated methods earlier than by the treating physician? A retrospective case-control study - Extended Data. https://doi.org/10.5281/zenodo.3862325 ^29^

- Appendix 1 Dementia Code List.xlsx
- Appendix 2 List of feature names.xlsx
- Appendix 3 GP consultation full data.xlsx

Data are available under the terms of the Creative Commons Attribution 4.0 International license (CC-BY 4.0).

### Code availability

Source code available from: https://github.com/ASTRODEM/detectability

Archived source code at time of publication: https://doi.org/10.5281/zenodo.3863004 ^32^

License: GNU Affero General Public License version 3 (AGPL-3.0)
