# Supplementary material for: Could dementia be detected from UK primary care patients’ records by simple automated methods earlier than by the treating physician? A retrospective case-control study
Source: Wellcome Open Res. 2020 Jun 8;5:120. [Version 1] doi: 10.12688/wellcomeopenres.15903.1 (PMC7385545; doi:10.12688/wellcomeopenres.15903.1)
Supplement: Peer review history

# Peer review: Reviewer response for version 1

**DOI:** 10.21956/wellcomeopenres.17443.r38992

**Published:** 2020-07-27

**Authors:** Deborah E. Barnes

**Affiliations:** 1 University of California, San Francisco, CA, USA; 2 San Francisco Veterans Affairs Health Care System, San Francisco, CA, USA

This manuscript describes development and testing of an automated algorithm that uses information in patients’ primary care records to determine which patients are likely to develop dementia so that they can be diagnosed earlier in the disease process. The sample included >95,000 U.K. patients, half with dementia and half without dementia matched on age, sex, and general practice. The primary findings are that prediction models had relatively low discrimination (AUC: 0.55-0.65) two to five years prior to the diagnosis. Although discrimination was improved (AUC: 0.84) during the year prior to diagnosis, many of the elements that improved prediction suggested that the clinician was already aware of the patient’s declining cognitive status and beginning to work them up for dementia (e.g., memory loss codes, referrals to neurology, cognitive screening). This is a largely ‘negative’ study, finding that information in the primary care EHR may not help identify patients with dementia earlier. However, it is well-conducted, well-written and provides important information to further our understanding of the strengths and limitations of using EHR data to detect dementia.

 It would be interesting for the authors to expand the discussion of predictors. For example, smoking, CHD, hypertension and deafness are potentially modifiable/treatable; infections, UTI, and dressing of wounds could be markers of poor health; antidepressants, antipsychotics, and Z-drugs for insomnia could reflect treatment of dementia-related symptoms; and home visits, ER visits, hospital admissions, and did not attend could be markers of inadequate primary care.

Is the work clearly and accurately presented and does it cite the current literature?

Yes

If applicable, is the statistical analysis and its interpretation appropriate?

Yes

Are all the source data underlying the results available to ensure full reproducibility?

Yes

Is the study design appropriate and is the work technically sound?

Yes

Are the conclusions drawn adequately supported by the results?

Yes

Are sufficient details of methods and analysis provided to allow replication by others?

Yes

Reviewer Expertise:

dementia, risk factors, study design, prognostic models

I confirm that I have read this submission and believe that I have an appropriate level of expertise to confirm that it is of an acceptable scientific standard.

# Peer review: Reviewer response for version 1

**DOI:** 10.21956/wellcomeopenres.17443.r39335

**Published:** 2020-07-20

**Authors:** Soo Borson

**Affiliations:** 1 Psychiatry and Behavioral Sciences, University of Washington School of Medicine, Seattle, WA, USA; 2 Kaiser Permanente Medical Care, Lancaster, CA, USA

This study uses machine learning as a tool for detecting dementia earlier than primary care clinicians in the UK do. The paper was a model of clarity and elegance, and a pleasure to read.

 The findings are extremely useful to the field of dementia detection: a number of enhancement attempts are being made using machine learning, but none has the special advantages of the CPRD and its longitudinal consistency. Efforts in the US are impeded by heterogeneity in practice patterns, record systems, and absence of a longstanding, national effort to improve detection and care of dementia in primary care.

 Particularly important in the design is the selection of medical record entry elements that practicing clinicians thought might be important early markers of dementia onset. 

 One element I’d like to see in subsequent contributions from this group is dementia stage/severity at diagnosis in primary care - i.e. how ‘early’ did detection occur?

 Current thinking in the field - except in primary care - proceeds from the assumption that early detection is a good thing, yet the meaning of ‘early’ varies with the stakeholder, and very little actual evidence exists to support it. It would be very valuable to learn more about what prompts UK PCPs to identify cognitive impairment and take steps to investigate it - the evidence we have from qualitative studies  could be greatly enhanced by a big-data perspective.

Is the work clearly and accurately presented and does it cite the current literature?

Yes

If applicable, is the statistical analysis and its interpretation appropriate?

Yes

Are all the source data underlying the results available to ensure full reproducibility?

Yes

Is the study design appropriate and is the work technically sound?

Yes

Are the conclusions drawn adequately supported by the results?

Yes

Are sufficient details of methods and analysis provided to allow replication by others?

Yes

Reviewer Expertise:

Dementia detection; dementia care; primary care of dementia; health system reform

I confirm that I have read this submission and believe that I have an appropriate level of expertise to confirm that it is of an acceptable scientific standard.
